# Prosocial behavior and gender

**DOI:** 10.3389/fnbeh.2015.00088

**Published:** 2015-04-14

**Authors:** María Paz Espinosa, Jaromír Kovářík

**Affiliations:** ^1^Fundamentos del Análisis Económico and BRiDGE, University of the Basque CountryBilbao, Spain; ^2^CERGE-EI, A Joint Workplace of Charles University in Prague and the Economics Institute of the Academy of Sciences of the Czech RepublicPrague, Czech Republic

**Keywords:** gender, prosocial behavior, treatment effects, economic games, altruism

## Abstract

This study revisits different experimental data sets that explore social behavior in economic games and uncovers that many treatment effects may be gender-specific. In general, men and women do not differ in “neutral” baselines. However, we find that social framing tends to reinforce prosocial behavior in women but not men, whereas encouraging reflection decreases the prosociality of males but not females. The treatment effects are sometimes statistically different across genders and sometimes not but never go in the opposite direction. These findings suggest that (i) the social behavior of both sexes is malleable but each gender responds to different aspects of the social context; and (ii) gender differences observed in some studies might be the result of particular features of the experimental design. Our results contribute to the literature on prosocial behavior and may improve our understanding of the origins of human prosociality. We discuss the possible link between the observed differential treatment effects across genders and the differing male and female brain network connectivity, documented in recent neural studies.

## Introduction

Prosociality is defined as any voluntary behavior intended to benefit other people and economic games have proven useful tools to learn about the nature of social preferences and motivations behind this behavior (e.g., Fehr and Fischbacher, 2003). Different variations of the benchmark games have been tested in the laboratory and in the field to analyze the determinants of human prosocial behavior and how it varies across different socio-economic contexts (Camerer, 2003) and social framings (Carpenter et al., 2003).

This study focuses on the role that gender plays in prosocial behavior. The experimental evidence on gender differences in social preferences is mixed (see Camerer, 2003; Croson and Gneezy, 2009, for reviews) However, rather than analyzying whether one sex is more prosocial than the other, as it is standard in the literature (Croson and Gneezy, 2009), we present evidence on gender differences on *how males and females react to treatment variations* (differential treatment effects, hereafter). To this end, we revisit several existing experimental data sets containing at least one treatment manipulation, and analyze gender-specific treatment effects with a special focus on two aspects of the experimental design, social framing and reflection enhancement. The original studies either find aggregate treatment effects on prosocial behavior or not and, although they generally control for gender within treatments, they do not typically explore differential treatment effects across the two sexes. This is the objective of our study.

Our hypothesis is that the mixed results on gender effects may have to do with the fact that different framing and conditions of the experiment may affect male and female subjects differently. In particular, we hypothesize that when the details of the experiment are put into a more “social frame”, female subjects will increase their prosociality with respect to a neutral baseline more than males, and that, when the frame allows or primes reflection, men will adjust their behavior toward their self-interest more than women.

The first hypothesis is suggested by the evidence that females react more than men to social and emotional stimuli in many contexts (see e.g., Brody and Hall, 1993, or McManis et al., 2001, among many others). Given this evidence, we expect this phenomenon to extend to social dilemma-like situations. This is consistent with Ellingsen et al. (2012, 2013) who run a Prisoners’ Dilemma experiment labeling the game either as “*Community Game*” or “*Stock Market Game*.” They observe that men and women are not different under the *Stock Market* frame. The *Community* treatment increases cooperation with respect to *Stock Market* but the effect is solely driven by the female participants. However, none of these two frames can be considered a neutral, context-free benchmark as each of them may induce a particular social norm.

As for the second hypothesis, the ability to regulate emotions impacts one’s social relationships (Gross and John, 2003) and large differences across males and females have been detected in this domain (e.g., McRae et al., 2008). Since emotions are naturally linked to social dilemma-like situations, the capacity to control emotions may be associated with lower sharing and less cooperation in these situations. In particular, men might be more able to abstract from the social and emotional aspects of social dilemmas and behave more in line with their self-interest if prompted. Several studies have analyzed how enhancing intuitive and reflective decision making affects behavior in social contexts (Rand et al., 2012, 2014; Rand and Nowak, 2013), but little is known as to whether such enhancement affects men and women differently.

The data confirm that women react more than men to social stimuli in these games and men behave closer to self-interested predictions with reflection and experience, compared to neutral control treatments. Regression analysis reveals that these differential treatment effects across men and women are generally significant in the studies that allow for within-subject comparisons but, even though they never go in the opposite direction, these differences are not robustly observed in our between-subject comparisons using the differences-in-differences approach.

## Materials and Methods

Previous literature has explored how experimental conditions affect prosocial behavior in a large variety of experimental games. This section introduces the economic games analyzed in this study, summarizes the data, and describes the statistical methodology applied to these data.

### Economic Games

We analyze data from three economic games:

**Dictator Game (DG)**. In the Dictator Game (DG), one player, the Dictator, proposes a division of a fixed amount of money between herself and another participant, the Recipient. Since the Recipient cannot but accept the proposed division, the amount given by the Dictator is considered an indicator of prosocial behavior. In the experiments, people on average give positive amounts of money to the Recipients but proposing zero is not uncommon (Engel, 2011). Women give more than men in some studies but gender effects are not robustly observed (Croson and Gneezy, 2009). Regarding social aspects of the game, reducing the Dictator–Recipient social distance and enhancing feelings toward the Recipient typically increase giving (e.g., Eckel and Grossman, 1996; Hoffman et al., 1996; Bohnet and Frey, 1999; Brañas-Garza, 2006; Brañas-Garza et al., 2012).

**Ultimatum Game (UG)**. The Ultimatum Game (UG) introduces one important modification to the DG. One player, the Proposer, proposes a division of a fixed amount of money between herself and another player, the Responder. In contrast to the DG, the Responder observes the proposed division and can either accept or reject it. If accepted, the money is divided as proposed; if rejected, both players earn zero. Hence, the Responder has a possibility to “punish” the Proposer if the former views the proposed division as unfair. The key difference between the Proposer and the Dictator is that the decision of the latter can be considered an indicator of her prosociality, while the former’s proposal confounds prosociality with strategic concerns. Consequently, we focus on the behavior of Responders. Self-interested individuals accept any amount and the more fair-minded a Responder is, the more likely she is to reject unfair divisions, with the rejection likelihood decreasing by the amount proposed for the Responder. In fact, offers below 30% are commonly rejected in experiments with human subjects whereas offers above 40% of the pie are rarely rejected; gender effects are not consistent across studies (Camerer, 2003; Güth and Kocher, 2014).

**Public Good Game (PGG)**. Public Good Game (PGG) is a continuous multi-player version of a social dilemma. There is a group of *n* players and each of them is endowed with the same amount of money. The experimental subjects have to decide how much of this amount they will hold in their private account and how much they will contribute to the public good. The money contributed to the public good is multiplied by a factor larger than one but lower than *n*, and placed into a public account. The payoff of each player in the game is the sum of her private account and an *n*-th part of the balance in the public account. Hence, the selfish choice is to keep all the money in the private account. If everybody does, the payoff of each player equals her endowment. The efficient outcome is achieved if all contribute the entire amount. The fraction of the endowment contributed to the public good serves as a measure of social preferences. People typically contribute positive amounts in one-shot PGG, with the average being slightly above 50% of the endowment; a non-negligible fraction of subjects never contribute anything (Camerer, 2003; Chaudhuri, 2011).

### Data

We focus on two types of experimental manipulations. Section Results: Prosocial Behavior and Social Framing reports studies that strengthen the social aspects of the games by introducing social framing; Section Results: Prosocial Behavior and Reflection analyzes experiments that promote (or inhibit) reflecting about the decisions.

#### Study 1

Brañas-Garza et al. (2010) conduct a DG experiment under three different treatments. In the benchmark, the Dictator proposes a division between herself and an anonymous stranger (*Neutral* treatment, *N* = 26, 16 females). Under one framing, subjects share the money with one of their friends in a previously elicited social network (*Friends* treatment, *N* = 27, 17 females). In a third treatment, subjects are told in the experimental instructions that the recipient “*relies on you*” (*Framing* treatment, *N* = 26, 17 females).

#### Study 2

Dreber et al. (2013) look at the effect of several types of framing in the DG. We focus on their Study 3 that provides the framing manipulation we are interested in and contains the largest number of observations (see the original paper for details concerning the other studies). We only consider donations lower or equal to 50%. The fraction of hypergiving (giving more than 50% of the endowment) is surprisingly large in their study and contrasts starkly with the typical distribution in the literature (Camerer, 2003; Engel, 2011). This selection results in 663 observations (291 females). Their two treatments differ in whether the Recipient knows that her payoff comes from a decision by another person (*Recipient informed* treatment, *N* = 327, 145 females) or not (*Recipient not informed* treatment, *N* = 336, 146 females). An important feature is that the Dictators are informed about whether or not the Recipient knows about the game. This is the framing we focus on here. Dreber et al.’s study contains a second framing variation by labeling the game as either *Giving* or *Keeping game*. As Dreber et al. have also admit, whether this framing is social hinges on subjects’ interpretations.

#### Study 3

Grimm and Mengel (2011) analyze the effect of delay on Responders’ decision in the UG. They conduct three treatments varying the timing of the decisions. First, they check whether imposing a delay, implemented by forcing participants to first answer a questionnaire immediately after observing the proposal and before making their decision (*Delay*, *N* = 132, 26 females), changes their acceptance rate with respect to the standard order, in which people decide right after observing the proposal (*No Delay* (*N* = 84, 19 females). In another treatment (*Change, N* = 126, 24 females), subjects respond right after learning the offer (as in the *No Delay* treatment) but, once they finish filling up the questionnaire, they are allowed to change their decision (as in the *Delay* treatment). In this case, each Responder makes two decisions: whether to reject right after observing the proposal and whether to reject after filling up the questionnaire, and this allows for within-subject comparisons.

#### Study 4

Brañas-Garza et al. (2013) conduct a repeated DG. They ran a DG experiment in October 2010 and repeated it in May 2011 with the same group of subjects (*N* = 199, 87 females in 2010; *N* = 163, 74 females in 2011; *N* = 136, 62 females in both). This design is suited to test how people change their behavior in the DG with experience and time delay and, as in Grimm and Mengel (2011), allows for within-subject comparisons.

#### Study 5

Rand et al. (2012) run a number of variations of the PGG to analyze how time pressure and time delay affect the contributions to the public good. Our interest is on the enhancement of reflection through delaying the decision. The *Benchmark* treatment (*N* = 212, 88 females) is the standard PGG, without any manipulation of timing, played on-line on the Amazon Mechanical Turk platform (Rand, 2012). This *Benchmark* treatment is compared to two variations: *Time Pressure* and *Time Delay*. Under *Time Pressure*, the participants are instructed to choose their contribution in less than 10 s; under *Time Delay*, they are instructed to decide after 10 s elapse. However, due to the typical lack of control of subjects in on-line experiments, many subjects disobeyed the time constraints. Rand et al. (2012) only consider the subjects who obeyed the constraints in their main analysis. For comparability purposes, we first follow their approach, leading to 194 (92 females) and 249 (97 females) observations in the *Time Pressure* and *Time Delay* treatments, respectively. We also repeat the analysis including the subjects who did not obey the time constraint (*N* = 372, 169 females and *N* = 308, 121 females, respectively).

#### Study 6

In another comparison, Rand et al. (2012) contrast the standard PGG with four treatments in which they either prime intuitive decisions or reflection. More precisely, before playing the PGG as in the benchmark, subjects see a screen, in which they are required to recall and describe in one paragraph a real-life situation, in which they decided on the basis of their intuition or reflection and whether the decision led to a good or bad outcome. The four treatments are: (i) intuition-good; (ii) reflection-bad; (iii) intuition-bad; and (iv) reflection-good. Since the first two treatments prime the intuitive decision-making whereas the last two prime reflection before a decision is made, Rand et al. aggregate the first two as the *Intuition-Priming* treatment (*N* = 175, 108 females) and the last two as *Reflection-Priming* (*N* = 168, 98 females). We follow their approach.

### Statistical Methodology

In each study, we analyze: (i) how the treatment manipulation affects each gender separately; and (ii) whether any detected gender differences are statistically significant. For objective (i), we disaggregate the data by gender and compare, separately for each gender, whether the behavior under the treatment differs from the behavior in the benchmark. We run two-sided non-parametric Wilcoxon rank-sum tests (also known as Mann-Whitney two-sample tests) if two different samples are compared and two-sided non-parametric Wilcoxon matched-pair tests when the behavior of the same people under two different treatment variations is contrasted. To address (ii), we use the standard differences-in-differences approach. We pool the control and treated observations and regress behavior on a female dummy, a treatment dummy (equal to zero in the benchmark treatments), and their interaction. Standard errors are robust to heteroskedasticity and clustered on subject when applicable. The estimation technique differs depending on the nature of the dependent variable (linear, ordered logit, and logit regressions). We detect a differential treatment effect across genders if the interaction term is significantly different from zero (Angrist and Pischke, 2008). Controlling for individual heterogeneity has no effect on the reported estimates.

## Results: Prosocial Behavior and Social Framing

### Study 1 (Brañas-Garza et al., 2010)

**Figure 1:**
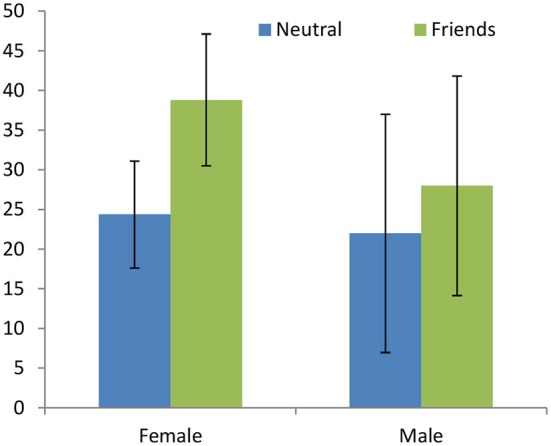
**Giving in the Dictator Game (DG; data: Brañas-Garza et al., 2010)**. Reducing the social distance between the Dictator and the Recipient affects females but not males.

Brañas-Garza et al. report that subjects are more altruistic when sharing with friends than with strangers (*p* = 0.013). Figure 1 disaggregates the average giving by male and female subjects in the *Neutral* and *Friends* treatments, revealing that only women react strongly to the treatment effect; men also increase their giving but the effect is not significant (*p* = 0.006 for female; *p* = 0.486 for male). However, men and women adjust their behavior in the same direction; the estimated difference in the treatment effect between the sexes goes in the correct direction but is not significant in the ordered logistic regression (*N*= 53, *p* = 0.338).

**Figure 2:**
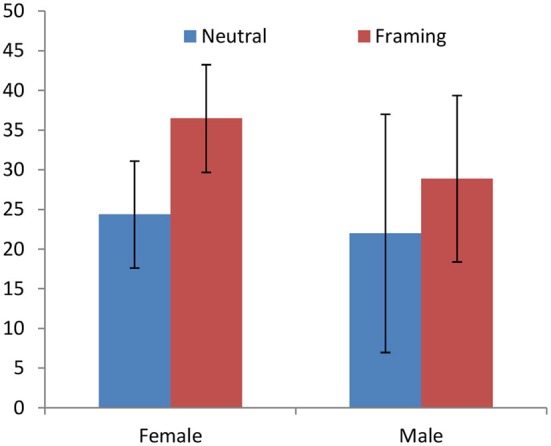
**Giving in the Dictator Game (data: Brañas-Garza et al., 2010)**. *Framing* (“*relies on you*”) affects females but not males.

The same type of result is obtained in the *Neutral*-*Framing* comparison. The overall treatment effect is significant (*p* = 0.015) but, as illustrated in Figure 2, it is mostly driven by women (*p* = 0.0073 for female; *p* = 0.4297 for male). The differences in differences are again not significant in the ordered logistic regression (*N* = 52, *p* = 0.431). Aggregating both treatments (*Friends* and *Framing* vs. *Neutral*) confirms no differential treatment effect (*N* = 79, *p* = 0.337).

### Study 2 (Dreber et al., 2013)

**Figure 3:**
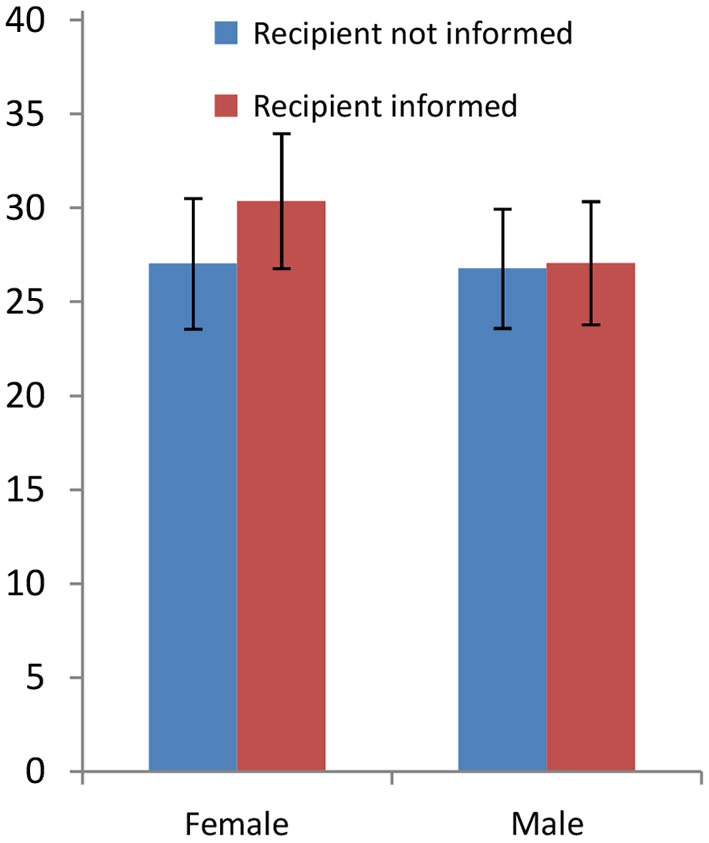
**Giving in the DG under two frames: Recipients are either not informed where the money comes from or informed that it comes from a game**. The condition was known by the Dictator (Dreber et al., 2013). Women seem to react more to the treatment effect, but the effects are never significant.

Dreber et al. find no effect on Dictators’ behavior of whether or not the Recipient is informed and the Dictator knows that (*p* = 0.359). Although women increase their giving more that men when Recipients are informed (see Figure 3), the gender-specific effects are not significant (*p* = 0.2010 for female; *p* = 0.9109 for male). The OLS estimate of the differential treatment effect is not significant (*N* = 663, *p* = 0.378). Despite never being significant, all these effects are in the expected directions.

The second framing variation in Dreber et al. (2013) tests the effect of labeling the game as either *Giving* or *Keeping game*. The aggregate effect is not significant (*p* = 0.928); women again seem to increase their giving more that men under the *Keeping* frame but the changes are not significant (*p* = 0.482 for female; *p* = 0.652 for male). The differential treatment effect is also not significant (*p* = 0.422). As mentioned above and noted by Dreber et al. the prosocial interpretation of this frame (*Giving* vs. *Keeping*) is disputable though.

To summarize, when the experimental design includes a change in the social distance between the Dictator and the Recipient or enhances the social nature of the game, the presented evidence suggests that women seem to react to social or emotional cues while male reactions are not statistically significant, but the difference between the two sexes is not statistically significant when applying the differences-in-differences approach. We could thus conclude in terms of Croson and Gneezy (2009) that “*the social preferences of women are more situationally specific than those of men*.” However, as we shall see below, this is not always the case; other design elements trigger large responses in men but not in women.

## Results: Prosocial Behavior and Reflection

### Study 3 (Grimm and Mengel, 2011)

**Figure 4:**
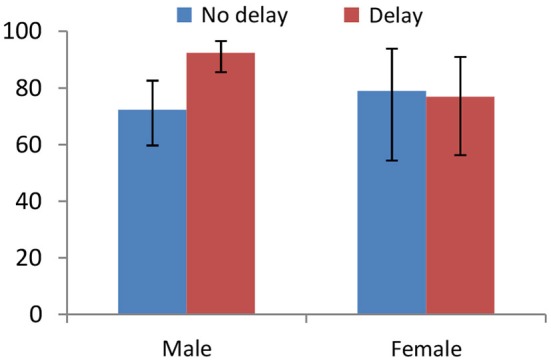
**The acceptance rate of all proposals by Responders in the Ultimatum Game (UG) for the *Delay* and *No Delay* treatments (Grimm and Mengel, 2011), disaggregated by gender**. The treatment effect is only significant for males, independently of whether we consider all or only low proposals.

Grimm and Mengel observe that a 10-min delay in the decision as to whether to accept or reject in the UG significantly increases the acceptance rate from 73.8% to 89.4% (*Delay* vs. *No Delay*: *p* = 0.003). Figure 4 uncovers that the treatment effect is only driven by male subjects (*p* = 0.001); women are virtually unaffected by the delay (*p* = 0.873). This is robust to only considering offers below 30% or 40% of the pie. Furthermore, the differences-in-differences logistic regression reveal that this difference between men and women is statistically significant (*N* = 216, *p* = 0.054).

**Figure 5:**
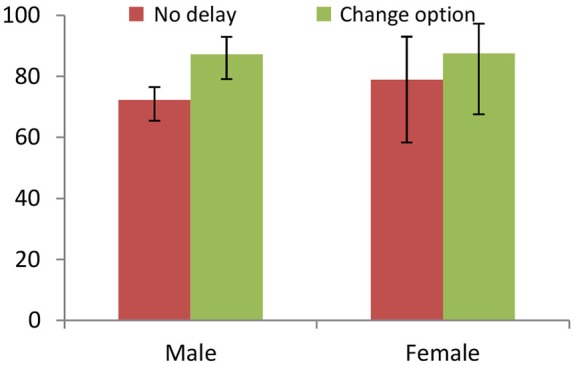
**The acceptance rate of proposals by Responders in the Ultimatum Game (UG) for the no delay and change treatments (Grimm and Mengel, 2011), disaggregated by gender**. The treatment effect is only significant for males and robust to considering low offers.

In the same vein, rejections are lower in the *Change* treatment with respect to the *No Delay* baseline overall (*p* = 0.013) but the effect is driven by males only (see Figure 5). For men, the fraction of accepted offers rises with respect to the baseline when allowed to change their decisions (*p* = 0.016), while the treatment differences are not significant in women (*p* = 0.456). The results are again robust if we restrict the analysis to low offers. In this case, the differential treatment effects are not significantly different across genders (*N* = 210, *p* = 0.715).

Comparing the two decisions in the *Change* treatment (within-subject), there is significantly less rejection after the 10-min delay needed to fill up the questionnaire (*p* = 0.001). The gender-specific tests show that men change their decision considerably while women do not (*p* = 0.007 for male, *p* = 0.180 for female). The aggregate effects remain significant for offers below 40% (*p* = 0.031) but disappears in the gender-specific comparisons (*p* = 0.109 for male, *p* = 0.289 for female). Since we compare behavior of the same subject, we regress a dummy for people that changed their decision from reject to accept on a constant and a female dummy in order to analyze whether males and females react differently to the chance of changing their decision. The estimated coefficients uncover that men react more than women to the change option (logistic regression, *p* = 0.085); this effect is stronger for offers below 40% of the stake (*p* = 0.040).

### Study 4 (Brañas-Garza et al., 2013)

Previous experience with a game allows for further reasoning about behavior and for potential learning. Brañas-Garza et al. design a study, in which people play the DG twice. In line with the above evidence, we hypothesize that the time elapsed between both games may affect men more than women.

**Figure 6:**
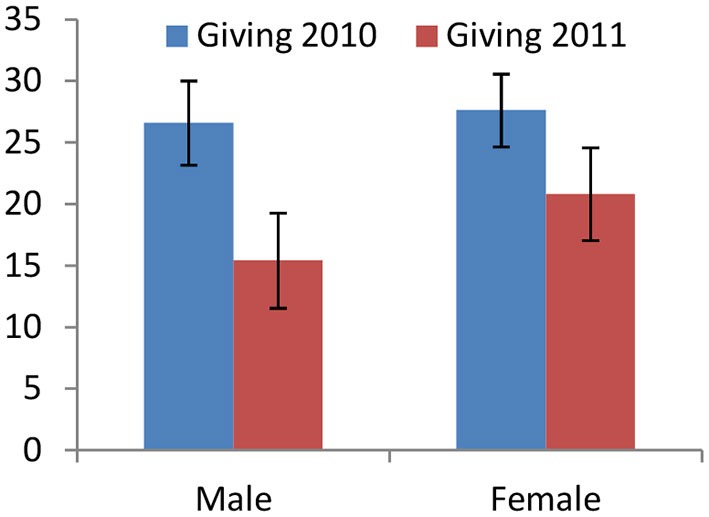
**Giving in the Dictator Game by the same subjects in 2010 and 1 year later (Brañas-Garza et al., 2013), disaggregated by gender**. Even though both genders decrease significantly their giving, men adjust their behavior more than women.

Overall, people decrease their sharing from the first to the second repetition (see Figure 6). The aggregate and gender-specific effects are significant (*p* < 0.0001 in all cases). However, the adjustment is stronger for men than for women: no gender difference in giving exists in 2010 (*p* = 0.8030), while 1 year later women share more than men (*p* = 0.0164). We conduct panel-data analysis to analyze carefully the differential treatment effects: the difference between genders is not significant in the whole sample (*N* = 225, *p* = 0.198) but regressions restricted to subjects who did not increase their giving (over 91%) reveal a differential treatment effect (*N* = 209, *p* = 0.024).

### Study 5 (Rand et al., 2012)

**Figure 7:**
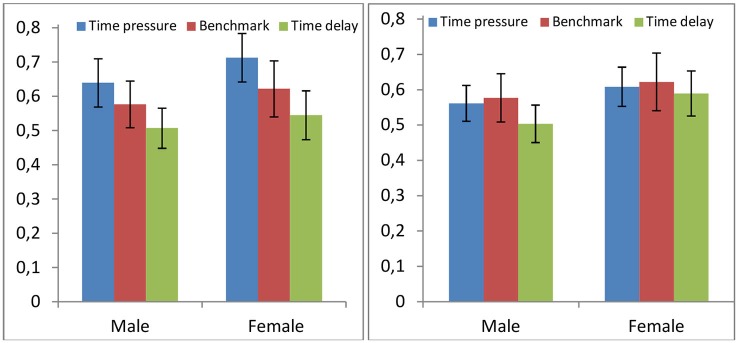
**Average contribution in the Public Good Game (PGG) under three differing conditions**. *Time Pressure*, *Benchmark* (without any time restriction), and *Time Delay* (Rand et al., 2012). Left: only subjects obeying the time constraint; Right: all subjects.

People give more under time pressure than in the standard PGG and more in the latter case than under time delay in Rand et al. (*Benchmark* vs. *Time Pressure*: *p* = 0.058; *Benchmark* vs. *Time Delay*: *p* = 0.028). We disaggregate their data by gender and graph the average contributions (%) to the public good in Figure 7, left. In case of the time obeying subjects, we observe no treatment difference either for men or for women between the *Benchmark* and any of the two variations (*Benchmark* vs. *Time Pressure*: *p* = 0.281 for males, *p* = 0.138 for females; *Benchmark* vs. *Time Delay*: *p* = 0.114 for males, *p* = 0.120 for females). The comparisons between the *Time Pressure* and *Time Delay* conditions are statistically significant on aggregate (*p* < 0.0001), for men (*p* = 0.008) and for women (*p* = 0.001). Figure 7, left, illustrates that men contribute somehow less than women but the differences are statistically weak (see Rand et al., 2012 for an exhaustive analysis). The interactions between the treatment (*Time Pressure* or *Time Delay*) and gender are never significant, showing no differential treatment effects (*p* > 0.4). This evidence does not support our working hypothesis but does not go against it.

In contrast, if we consider all the subjects and not only those who obey the time constraints, the results are in line with our hypothesis; see Figure 7, right. The *Time Delay* treatment is different from the *Benchmark* on aggregate (*p* = 0.056), mostly due to men’s behavior (*p* = 0.078 for male, *p* = 0.385 for female). The results in the *Time* *Pressure* treatment are not different from the *Benchmark* (*p* > 0.56 for all cases). The differences in how men and women react to the treatments are never significant (*p* > 0.4) However, since we cannot tell why people do not obey the time constraint or whether those who do not are different from those who do, it is difficult to interpret the differences between these results and those that exclude the time-disobeying subjects.

### Study 6 (Rand et al., 2012)

**Figure 8:**
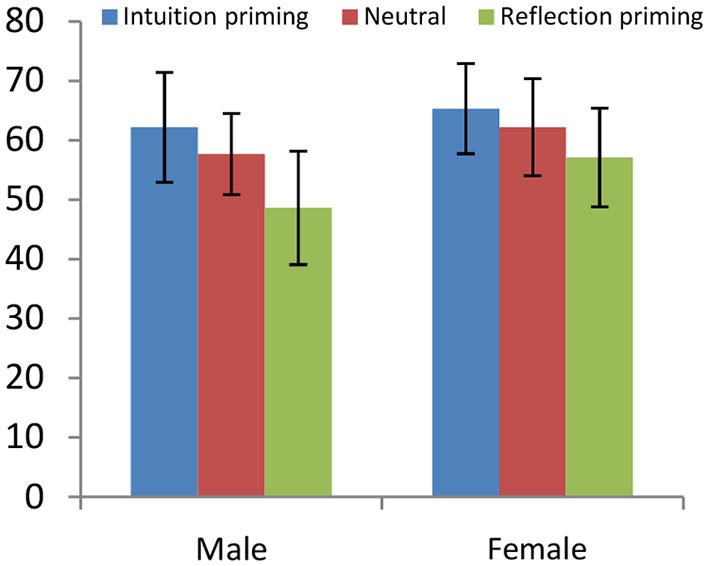
**Average percentage contribution in the PGG under three differing conditions: *Intuition Priming*, neither intuition nor reflection are enhanced (*Benchmark*), and *Reflection Priming* (Rand et al., 2010).** No gender is affected by *Priming Intuition*; only men react to *Reflection Priming* significantly decreasing their contributions.

As for the comparisons between the *Intuition-Priming* and the *Reflection-Priming* treatments to the baseline, the difference between the *Benchmark* and *Intuition-Priming* is significant for no sex (*p* = 0.4879 for males, *p* = 0.4775 for females) and the difference between the* Benchmark* and the *Reflection-Priming* treatment is weakly significant for males (*p* = 0.0922) but not for females (*p* = 0.3806). The comparison between *Intuition-Priming* and* Reflection-Priming* is significant for males (*p* = 0.0489) but not for females (*p* = 0.1063). These results are in harmony with the above evidence that men but not women react to reflection enhancing. In the regression analysis, the interaction between gender and the treatment is never significant (*p* = 0.545), suggesting no differential treatment effects (see Figure 8).

Overall, the data in this section suggest that allowing or priming reflection in social decision-making decreases prosociality and cooperation in men but less in women. This contradicts the common view in experimental economics that male exhibit more stable behavior in social games than women (Croson and Gneezy, 2009; Miller and Ubeda, 2012) and calls for a reformulation of the intuition-cooperation linkage proposed by Rand and his colleagues (Rand et al., 2012, 2014; Rand and Nowak, 2013). These issues are discussed further in the next section.

## Discussion

We find some discrepancies across men and women in the way they react to design manipulations. In particular, female social behavior tends to be more affected by social and emotional aspects of the experimental design, whereas men tend to adjust their behavior more than women when subjects are motivated to reason further about their behavior. This is reflected in that only one gender generally reacts significantly to the treatment in the data separated for men and women. The tests of different reactions to treatment variations across genders are supported in data sets that allow for within-subject comparisons (Grimm and Mengel, 2011; Brañas-Garza et al., 2013) but the effects are statistically weak in our between-subjects comparisons applying the standard differences-in-differences approach. Consequently, we view the present evidence as indicative and we stress that more research on these issues is necessary.

Notwithstanding this, this study reveals that gender is an important element of human prosociality as the mechanisms stimulating or inhibiting social behavior seem to differ across male and female subjects. This should not be a surprise, since their social roles have differed for the most part of human history and, depending on the social context, different behaviors are expected from men and women in virtually all cultures around the Globe (Eagly, 2013). If men and women follow different norms for behavior in different social contexts, we are likely to observe similar differences in the laboratory when there is a framing that subjects can associate to the corresponding social situation (see Carpenter et al., 2003).

One implication of our study is that women, rather than being more prosocial, may appear more prosocial in some studies as a result of the details of the experimental design (even if the manipulation only affects males), but both genders behave equally when the context is experimentally neutral, deprived of a frame. These findings point to the crucial role of gender, beyond the simple comparison of male and female behavior within treatments, and suggest that the role of gender should be investigated further.

The present evidence supports Croson and Gneezy (2009) in that women care more about the social context. However, our results contrast with their main conclusion and the common belief in the experimental literature summarized by Croson and Gneezy (2009, p. 1): “*Social preferences of women are more situationally specific than those of men; women are neither more nor less socially oriented, but their social preferences are more malleable*.” The current paper illustrates that the social behavior of both genders is malleable, but each responds to different details of the context. Females react more to aspects of social framing whereas males are more affected by reflection-related manipulations.

We also contribute to the literature initiated by David Rand and his coauthors (Rand et al., 2012, 2014; Rand and Nowak, 2013) who argue that intuition enhances cooperative behavior and reflection inhibits it. They hypothesize that people internalize behavioral norms which are advantageous in their daily life situations and apply them in atypical situations such as lab experiments. Only when prompted to reason or reflect about the new situation or in-lab experience, experimental subjects behave more in line with their self-interest. To support this hypothesis, this literature manipulates the timing of the decisions in social dilemmas and looks at the differences between experienced and inexperienced experimental subjects. Their data largely support this hypothesis (see Rand et al., 2014). Our evidence points to a potential interaction between gender and this reflection-cooperation linkage. See also Kahneman (2011) or Rubinstein (2007) for different arguments regarding the timing of a decision.

The question is whether the detected behavioral differences between men and women can be explained. To this end, we appeal to social neuroscience, a field integrating neuroscience, cognitive and social sciences (Cacioppo, 2002; Han and Northoff, 2008). Of particular interest is the recent research on the structural connectome, the neural connectivity of the human brain. The pattern of interconnections is a determinant of how the global network, i.e., the brain, works (Hagmann, 2005; Sporns et al., 2007; Bassett and Gazzaniga, 2011). Importantly, recent research detected significant discrepancies in inter and intra-hemispheral brain connections across genders (Gong et al., 2009; Tomasi and Volkow, 2012; Ingalhalikar et al., 2014; but see also Dennis et al., 2013). In a sample of 949 subjects, Ingalhalikar et al. (2014) report that males exhibit higher intrahemispheric cortical connectivity than females, while female brains display higher interhemispheric and modular connectivity. They also find higher modularity and transitivity (network measures reflecting how easily a network can be divided into subnetworks) in males. Based on this evidence, they conclude that female brains are better adapted to facilitate the “communication” between the modules of the brain as well as the analytical and sequential reasoning modes of the left hemisphere and the intuitive processing of information of the right hemisphere, whereas males’ within-hemispheric supratentorial connectivity would enhance coordinated action (see also Cahill, 2014). Indeed, in an earlier behavioral study that included the same experimental subjects of Ingalhalikar et al. (2014), females outperformed males on attention, word and face memory, and social cognition tests, while males performed better on spatial processing and motor and sensorimotor speed (Gur et al., 2012; Roalf et al., 2014; Satterthwaite et al., 2014). Thus, consistent with the arguments of Ingalhalikar et al. (2014), certain behavioral and cognitive gender differences may be traced back to the differing patterns of interconnectedness of male and female brains.

We hypothesize that the structure of the human connectome could be related not only to cognitive and perceptual processes, but also to social preferences and prosocial behavior. More precisely, the nature of the detected differences in social behavior of men and women might be associated to women’s higher cross-module and interhemispherical connectivity that facilitates the integration of modular functions. It has been largely documented in neuroeconomics that different brain modules are associated e.g., with negative emotions (for example, anterior insula) and other brain areas are responsible for cognitive control of emotional reactions (such as different parts of the prefrontal cortex); Glimcher and Fehr (2013) provide an excellent exhaustive review of this literature. Denser wiring among modules and lobes in women may stimulate the simultaneous involvement of these modules in social decision-making. This could be behind women’s larger responsiveness to social frames. Lower cross-module connectivity in men, in contrast, may predispose them for easier “disconnection” of modules, explaining partially why they can look less other-regarding if they take time resolving the inner conflict between selfishness and fairness. In fact, an asymmetry across sexes in brain activation has already been observed in social contexts (Singer et al., 2006).

These arguments are in line with Bullmore and Sporns (2012) who propose that the architecture of the human brain network is shaped by a trade-off between the biological costs and the efficiency of the pattern of anatomical connectivity in terms of its adaptive value (see also Niven and Laughlin, 2008; Bassett et al., 2010). We propose that this reasoning may also govern the proper functioning of the brain and that such evolutionary criterion may lie behind the gender-specific neural circuitry. Women’s higher interhemispheric connectivity may have evolved to allow for cheaper involvement of modules and brain lobes in terms of biological costs while making decisions, and complicates the ability to disconnect them, whereas male brains seem to be better adapted to overcoming the conflict between individual reward and other-regarding concerns.

Even though our results are consistent with these ideas, this study can by no means be considered direct evidence of the relationship between performance and brain connectivity. This document seeks to stimulate future research that would test whether such a direct link exists. Many neuroscientists indeed call for further analysis of the brain-behavior linkage (e.g., de Vries and Södersten, 2009; Biswal et al., 2010). The presented evidence particularly calls for the link between the architecture of the structural connectome and behavior in social games. Such data would largely enhance the understanding of the underpinnings of social preferences and human cooperation.

## Conflict of Interest Statement

The authors declare that the research was conducted in the absence of any commercial or financial relationships that could be construed as a potential conflict of interest.
